# The Treatment of Chronic Constipation: A Discussion at the Bristol Medico-Chirurgical Society, February 12th, 1913

**Published:** 1913-03

**Authors:** F. H. Edgeworth


### THE TREATMENT OF CHRONIC CONSTIPATION.

A DISCUSSION AT THE BRISTOL MEDICO-CHIRURGICAL SOCIETY, FEBRUARY 12TH, 1913.

INTRODUCED BY

F. H. EDGEWORTH, M.D. Cantab., D.Sc. Lond.

THE contents of the small intestine are fluid in character, travel rapidly through the 22 feet of its length, and begin to issue through the ileo-cæcal valve within four to five hours of a meal. They travel through the large intestine far more slowly, reaching the hepatic flexure in six and a half hours, the splenic flexure in nine hours, and the pelvic colon in about eighteen hours—in a semi-solid condition owing to the absorption of water. Here the fæces are stored, losing still more water, until defæcation takes place. This reflex act, started by the entry of fæces into the rectum, and aided by voluntary contraction of the abdominal wall, empties the colon from the splenic flexure downwards.

The fæces chiefly consist of water (65%), cellulose and bacteria. Most of the proteid, fat, and sugar of the food is absorbed. The bulk of the fæces—on which so largely peristalsis depends—is mainly due to the, mostly undestroyed cellulose of the vegetable constituents of the food.

By constipation is understood a delay in the passage of the intestinal contents through the large intestine ; and it is convenient (following Hertz) to divide such cases into those in which delay occurs proximal to the pelvic colon, and those in which defæcation is defective. There is no good word to signify the former class of case, the latter is termed dyschezia. The occurrence of delay is easily ascertained by the administration of charcoal biscuits—the fæces are normally blackened within forty hours.

Ordinary constipation—in which delay takes place above the pelvic colon—may be due to one or more of many causes, *e.g.* to weakness of the intestinal wall as in old age, or to relative weakness when the fæces are hard and dry—when too little fluid is taken or too much absorbed, as in diabetes ; or it may be the result of insufficient mechanical and chemical stimulation of the intestinal wall, due either to absolute insufficiency of the food or—much more commonly—to a relative insufficiency of its cellulose contents. This creates a vicious circle—the deficiency in water or cellulose lessens the bulk of the food residues and slows their passage, so that a still greater quantity of water is absorbed, with resultant delay and difficulty in transit. Or reflexes may not occur, *e.g.* when the mucous membrane is damaged by an attack of diarrhœa, or when the nervous centres are inactive, as in neurasthenia and melancholia, or inhibited by some peripheral irritation, as by an inflamed ovary. The lumen of the bowel may be narrowed by localised spasm of its muscular coat. If this is acute the condition is generally mistaken for intestinal obstruction, if more chronic and less intense it may be recognised by discovery of contracted lengths of tender intestine which vary from day to day. The lumen may be narrowed by a new growth, not far enough advanced to give rise to intestinal obstruction with its attendant symptoms and physical signs. Such neoplasms, especially when they occur at the hepatic or splenic flexures, may cause the greatest difficulty in diagnosis in the initial stages. According to some authorities, peritoneal adhesions may narrow the lumen of the large intestine to a degree sufficient to cause constipation —as distinguished from obstruction. It is, however, unwise to suggest any operative interference unless skiagraphy after bismuth meals shows some positive localised delay in the passage of the intestinal contents.

If causes necessitating operation can be excluded, the following are the best means of treating the condition. Sufficient water should be taken. The supply of cellulose-containing articles of food should be augmented. Of these the most important are porridge, wholemeal bread, and cooked vegetables of all sorts. Fruits are apt to cause colic unless taken at the conclusion of a meal. Bananas are useless, owing to the small percentage of cellulose.

Of drugs the following are of use. Salines are most useful if the blood-pressure is high, if low they cause too much depression. In the former condition it will be found that salines not only act as purgatives but are most efficient vaso-dilators. Mercurials should not be given as a regular aperient in chronic constipation, and never if nephritis is present; in the latter condition one 5-grain dose of blue pill may set up severe ulcerative stomatitis. The only condition in which calomel is useful as a regular aperient is typhoid fever with constipation. Cascara is an excellent aperient, though a little apt to lose in efficiency in the course of months; it is best given in a pill; the liquid extract is very bitter. Aloin is probably the best aperient in chronic constipation, for it will be generally found that the dose can be gradually diminished. This is specially the case when pills containing only ¼-grain of aloin are given —the patient takes the necessary number (3—8) to produce one non-fluid action each day or every other day, and lessens the dose by one pill if more than one action or a fluid action results.

Senna is apt to cause colic. Rhubarb and castor oil act well, but their effects are followed by increased constipation; rhubarb, however, is a most useful drug for children with capricious appetites, with or without constipation; and castor oil is invaluable in the treatment of membranous colitis—in doses of half to one ounce every morning. I have had no experience with purgen, though some of my friends speak highly of it. Podophyllin may be employed alone in children with hard clayey stools, and may be added to any of the above purgatives if patients are dull and depressed or have a metallic taste in the mouth in the morning. Sulphur—5 grains in a lozenge—has the effect of keeping the motions soft, and is particularly good in old people: some, however, cannot take it owing to its causing offensive breath. Liquid paraffin has come into use during the last few years, in doses of half to one ounce once or twice a day; it is a most efficient laxative; for children it may be mixed with a little glycerine and a drop of peppermint oil; there is no advantage in ordering an emulsion. Agar-agar is also very good, it absorbs water and so prevents the motions becoming hard; it may be given in tablespoonful doses once a day, ground up in a coffee mill. Regulin (agar-agar mixed with cascara) presents no advantages over agar-agar, and is very nasty.

In spastic constipation ordinary purgatives do not act, and make the pain worse; they should be mixed with belladonna, or the latter drug given alone. This has the power of relaxing involuntary muscle, and so relieves the condition and acts as a laxative.

I now pass on to the other great variety of constipation, that of dyschezia, in which there is either difficulty or inability to empty the pelvic colon in defæcation. The condition is easily recognised—if the rectum be examined after defæcation has taken place it will be found to be full of fæces. At the same time the pelvic colon—examined through the anterior rectal wall—is found full, a sausage-like, indentible mass. The iliac colon, too, is often found full, and sometimes tender on pressure. The condition may be brought about in many ways. It may follow on ordinary constipation owing to the slowly-moving fæces becoming dry and hard. It often results from the pernicious habit of disobeying natural calls to stool, whereby the sensitiveness of the rectal mucous membrane is impaired or lost, and the normal reflex act set up by entry of fæces from the pelvic colon into the rectum does not occur; or there may be some narrowing of the rectum, either functional or organic in nature. Thus, an anal fissure or inflamed piles may make defæcation painful and bring about postponement of the act; or cancer may narrow the passage. Statistics show that four-fifths of all cases of cancer of the large intestine occur in the pelvic colon or rectum; and the only good rule to observe is that if a middle-aged or elderly person, without change of habits, begins to suffer from constipation or diarrhœa, a careful examination should be made, both by finger and sigmoidoscope. Apart from such cases the main cause of dyschezia is weakness of the abdominal wall, a most common cause in middle-aged women of the poorer class who have had many children, and in whom no attempt has been made by exercises after childbirth to restore its integrity. The voluntary contraction of the abdominal wall in defæcation is consequently inefficient, and the pelvic colon and rectum are incompletely evacuated. Such cases are also frequently the subjects of visceroptosis—an associated, not a causal, condition of the dyschezia.

The treatment of dyschezia, if not due to a surgical cause, is by enemata—purgatives are useless. At the same time regular habits of soliciting an action of the bowels should be inculcated. If the cause be weakness of the abdominal walls, these should be strengthened by massage and afterwards by voluntary exercise—" heads and tails " are excellent; in many cases, however, the abdominal wall is damaged beyond repair, and then a well-fitting abdominal belt should be worn. The motions should be kept soft by sulphur, paraffin, or agar-agar.

### DISCUSSION.

Mr. T. Carwardine discussed the causation and effect of adhesions which led to constipation. He alluded to the effect of chronic appendicitis. He had met with adhesions binding the terminal part of the ileum to the pelvic wall and cured them by separating the adhesions. One case he had found to be due to a retro-colic hernia. Those present who had treated cases by short-circuiting or colectomy were asked to relate their experiences.

Mr. F. G. Bergin emphasised the advantages obtainable by skiagraphy after bismuth meals. The help given in locating the nature of intestinal stasis by this method was enormous. The physicians and surgeons should themselves examine the cases with the screen. Skiagrams gave much less information than careful and prolonged watching with the screen. Cases illustrating the value of this method were reported.

Dr. Bertram Rogers discussed constipation as it affected infants and children. In such patients regular habits had not been acquired. Training should be given when possible. He considered cascara a good remedy, and the taste could easily be concealed with liquorice.

Dr. Michell Clarke said that the causes of constipation had been so fully and ably dealt with by the opener of the discussion that he should say very little on this head. The first steps in diagnosis must be to eliminate any general disease which might be the cause of constipation. The examination of a patient at any age suffering from constipation must include an examination of the rectum. This might seem too obvious for mention, were it not that in his experience it was often omitted, and sometimes with disastrous result from the overlooking thereby of some disease of the rectum or pelvis.

The large majority of cases of constipation were in women, and increasing experience had taught him that the most frequent cause was neglect of the call to defæcate, either from shyness, carelessness, or undue haste, in girls of the school age. Too much importance could not be attached to sufficient provision being made and time allowed for a morning evacuation of the bowels in girls and boys at school. History of cases of long-standing constipation in adults generally showed that the condition dated from this period of life, and it was not uncommon to obtain a further history of attacks of abdominal pain, sometimes severe and lasting for days, between the ages of eight and seventeen, which might be reasonably attributed to inflammatory lesions around the cæcum or in the appendix, and resulting in the formation of adhesions in this region.

Further, although exercise was undoubtedly a preventive of constipation, yet the present strenuous routine of life at school and subsequently at college, in which every hour of the day was occupied by brain work or games, and in which also the hours of sleep were often too short, might lead to nervous exhaustion and through this to constipation. Certainly some of the most obstinate cases of constipation he had had to treat occurred in youths and girls distinguished in athletic games. He fully endorsed all that Mr. Bergin had said as to the value of the X-rays with a bismuth meal in the diagnosis of constipation.

For the treatment of the ordinary case, arising from former neglect of the normal call to defæcation, the first point was to correct the diet, especially in the matter of taking sufficient water, fats, green vegetables and fruits. A glass of hot water should be taken on rising and going to bed. In all these food-stuffs the diet was apt to be insufficient, whilst there was an excess of tea and sweet-stuffs. The next was to get rid of drugs : these patients were generally addicted to taking the much advertised purgatives. It was, however, generally necessary to begiu treatment by an aperient of some kind, and perhaps one of the most useful was a pill of iron, aloes, nux vomica and belladonna given two or three times a day, the dose of which could be gradually reduced, and finally left off. One of the preparations of petroleum was a useful adjunct. Other measures were to insist on the patient's going to stool daily at a fixed hour by the clock, to see that the seat of the w.c. was not too high, and the carrying out of exercises which strengthen the abdominal muscles, as well as regular daily exercise in the open air for those leading a sedentary life.

The more severe cases, which were so often associated with neurasthenia, and in which constipation was due to nervous exhaustion, might generally be cured by a course of treatment in a nursing home, with a very full diet, rich in fats, green vegetables, fruit, etc., abdominal massage, the application of (sinusoidal), electrical currents, over the course of the colon, together with rest in bed at first, and suitable exercises during the latter part of the cure. Treatment must be initiated by a thorough cleaning out of the large bowel by large enemata of oil followed by soap and water. It was important to remember that when once enemata of this kind were begun they must be carried out completely, otherwise toxic absorption might occur, of which there was little danger so long as the accumulated fæces were undisturbed, for the body was well protected against this. Small enemata of cold water were useful subsequently, and seemed to stimulate the bowel. Very severe and long-standing constipation could be cured by such a course of treatment. Stercoral ulcers were in his experience rare, but were probably best treated by emptying the bowels and putting the patient on a full and nutritious diet.

Cases in which the symptoms pointed to adhesions around the cæcum or to chronic lesions of appendix would require surgical treatment.

Visceroptosis was a most important and not uncommon cause of constipation, and one often very difficult to treat; where medical measures after a full trial had failed, and in those cases in which the colon formed a large V-shaped loop descending to the pelvis the operation for short-circuiting the large bowel should be tried.

A dilated and dropped stomach might be present alone, and in such cases constipation was the rule, due to the very defective absorption of water: treatment directed to the stomach condition would be most likely to cure the constipation.

Lastly, no form of treatment would be permanently successful unless the patient afterwards paid careful attention to getting an action of the bowels at a fixed time every day and kept to a suitable diet.

Mr. J. Lacy Firth related an experience of surgical intervention for the cure of constipation in a woman of about 24 years of age. Prolonged medical treatment had failed. First appendicostomy had been performed in the hope of improving matters by washing out the colon. Some months later ileo-sigmoidostomy with division of the ileum below the anastomosis was performed, and the appendicostomy opening was still utilised for irrigation. The patient returned home, but after some months came back to hospital with the appendix-opening closed and the colon and cæcum full of impacted fæces. Finally, the entire colon down to near the sigmoid was removed. The patient was much better when last heard of some three months after the operation.

Mr. Lansdown emphasised the importance of rectal examination. He had been asked to operate upon cases in which such examination had been omitted, and when the cause of obstruction was a loaded rectum. If short-circuiting was done the anastomosis should be into the beginning of the rectum, not into the pelvic colon, otherwise the latter would act as a cesspool. He mentioned a case in which the cause of obstruction had proved to be a kink at the junction of the pelvic colon with the rectum. The bowel was washed out by means of a long tube for over an hour with the abdominal wound still open, and then the sigmoid attached to the anterior abdominal wall. There had been no trouble since.

The President reminded members that German rye bread was procurable in Bristol, and was a useful article of diet in constipation. Garlic and agar-agar he could also recommend.

